# Environmental scan of COVID-19 infection dashboards in the Florida public school system

**DOI:** 10.3389/fpubh.2022.925808

**Published:** 2022-07-29

**Authors:** Hye Ryeon Jang, Jordan Quinones-Marrero, Juan M. Hincapie-Castillo

**Affiliations:** ^1^Department of Political Science, College of Liberal Arts and Sciences, University of Florida, Gainesville, FL, United States; ^2^Department of Epidemiology, Gillings School of Global Public Health, University of North Carolina at Chapel Hill, Chapel Hill, CA, United States; ^3^Injury Prevention Research Center, University of North Carolina at Chapel Hill, Chapel Hill, CA, United States

**Keywords:** public health dashboards, COVID-19, public health surveillance, dashboard and visualization tool, public school

## Abstract

Public dashboards have been one of the most effective tools to provide critical information about COVID-19 cases during the pandemic. However, dashboards for COVID-19 that have not received a lot of scrutiny are those from the public school system. We conducted an environmental scan of published dashboards that report and track new COVID-19 infections in the Florida public school system. We found that thirty-four percent of counties do not provide any public dashboard, and there was significant heterogeneity in the data quality and framework of existing systems. There were poor interfaces without visual tools to trace the trend of COVID-19 cases in public schools and significant limitations for data extraction. Given these observations, it is impossible to conduct meaningful policy evaluations and proper surveillance. Additional work and oversight are needed to improve public data reported.

## Introduction

Public dashboards are important tools in communication efforts in public health and have been used extensively throughout the COVID-19 pandemic (1, 2). While highly prevalent, there is wide variation in the configuration of current dashboards and surveillance systems available to the public (3, 4). With the several mitigation policies implemented across all jurisdiction levels in the United States, these online platforms have become an important source of local case data for policy and personal decision-making. Limited research on best practices for implementations of actionable dashboards points to the need of presenting information that is responsive to the specific needs of the audience and utilizing storytelling and visual cues (5). There is no consensus on which audience should be a target of COVID-19 dashboards. While some experts argue that dashboards should provide public health information to the general public (3, 6–8), others point to the need to inform public health workers, researchers, and policy makers (2, 4, 9–11).

Ultimately, the specific end goals of presenting case data in each health dashboard will depend on unique motivations and mandates of its curators and administrators. Systematically assessing the features and capabilities of dashboards permits public health professionals to understand, and, ultimately, remediate the shortcomings of these surveillance platforms in meeting their specific goals. To this end, environmental scanning is a helpful analytical process of data gathering that identifies gaps in the needs of the end-users and the pitfalls of design and presentation of the information (12). Given the implications of publicly available surveillance systems in influencing both public behavior and regulatory action, a thorough review of the features available in COVID-19 case dashboards is a necessary task, albeit a time consuming one.

Dashboards for COVID-19 case data that have not received a lot of scrutiny are those from the public school system. Children were and continue to remain particularly vulnerable to the effects of the pandemic as a result of delays in vaccine availability (13). These issues are exacerbated in school settings where social distancing is challenging and cases arise when mitigation efforts fall short. Not having a comprehensive appraisal of COVID-19 public dashboards to inform residents in specific localities is concerning for public health outreach efforts. This gap in the literature is also significant given that throughout the pandemic, parents of school-aged children and school administrators have had to confront with constant decision-making on the best approaches to mitigate the spread of infections (14). These deliberations not only require access to accurate, current, and timely case data, but also necessitate having user-friendly electronic systems. Using the State of Florida public school as a case study, we aimed to systematically collect and appraise data of available COVID-19 public dashboards and discuss the advantages and shortcomings of these surveillance systems.

## Method

We conducted an environmental scan of online published dashboards that report and track new infections in the State of Florida public school system. Public schools in the State of Florida are administered by county school boards, thus, we manually surveyed the websites for each county school board in the State (*n* = 67 counties) up to the end of May 2022. Two reviewers extracted data independently and each discrepancy was reconciled through consensus. We performed a thematic analysis of their characteristics and their configuration, and the team critically appraised the dashboards using published literature on best practices for the creation and implementation of these systems. Data elements extracted for each available dashboard included variables on data quality (level of aggregation, timeline of case data, timeliness, and availability of metadata) and graphic quality (presence of visualization, ability to filter data, cleanliness). While there are important elements to evaluated in dashboards such as fidelity, reliability, sustainability, and transparency of data, we intentionally limited our scope to information available to the public through online access. We used ArcGIS Desktop 10.8.1. (Redlands, CA) to generate a map of available dashboards to assess the geographic distribution of counties across the state.

## Results

### Availability and features of public dashboards

**Figure 1:**
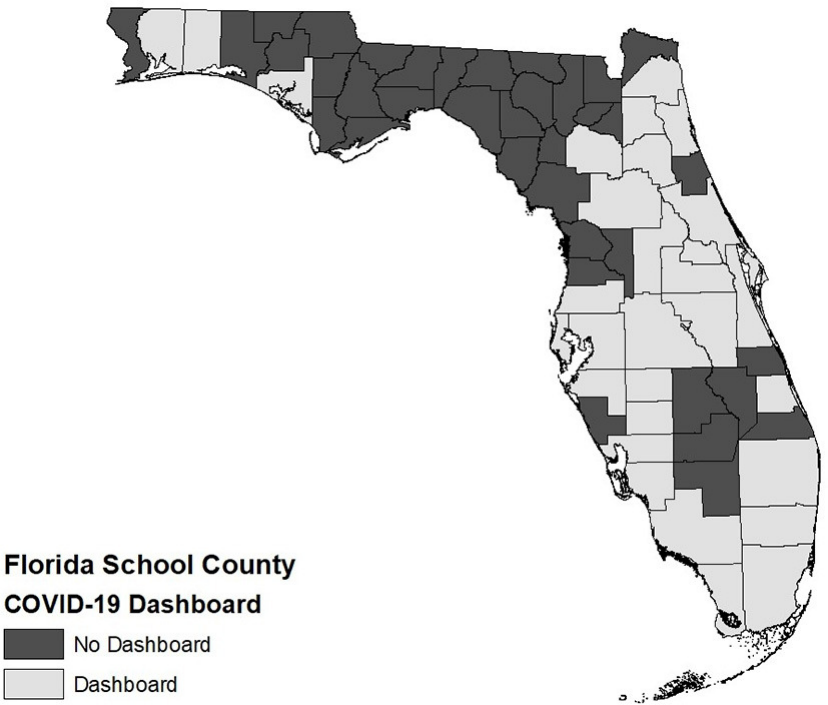
Availability of COVID-19 dashboards in Florida Public School Districts.

There were forty-four counties in Florida (65%) that provided a COVID-19 case dashboard for their public schools by February 1, 2022. As of May 31, 2022, twenty-nine county public school boards (43%) had a dashboard still available (Figure 1). To evaluate the quality of each dashboard in our thematic analysis, we appraised two common criteria of appropriateness from literature in public health and bioinformatics: data quality and graphic quality. We considered the public as well as policy makers and researchers as potential audiences when assessing these quality criteria.

Having valid and reliable data available for COVID-19 infections in public school dashboards is important for researchers and policy makers to understand current and emerging trends. Public dashboards should provide the most disaggregated-level data, so researchers can collect relevant information in any level of analysis. For example, dashboards should regularly update daily cases of students and staffs (separately) per school in the county. In addition, dashboards should provide a full timeline of COVID-19 cases since August 2021, which enables researchers to trace down the COVID-19 cases throughout the school year and conduct temporal trend analyses (3, 10, 11, 15). Moreover, it is important to provide metadata so the audience can understand how accountable the data is (16).

Graphic quality in county public school dashboards determines the accessibility of information to parents or local community members. First, dashboards should use an interactive graph to present the trend of the COVID-19 cases (3, 10, 17). Florida public school COVID-19 dashboards typically use Microsoft BI^®^ or Tableau^®^ to generate bar or line graphs to indicate trends in a visually appealing manner. This approach is efficient as it does not require users to review out-of-context raw numbers across multiple tables, to download single reports, or to link to another interface. Moreover, it is important to locate all essential graphic information on a single screen in a simple and concise way (3, 6, 9, 10). By doing so, dashboards can reduce cognitive processing of users and make them easily accessible. Finally, dashboards should allow users to apply filters into the graph to trim different timelines or specify region or location of schools.

### Challenges and gaps in surveillance

There are significant challenges for the existing platforms available in the Florida public school system (Table 1). First, many dashboards do not provide disaggregated-level data due to the lack of a unified standardized platform in Florida's county school boards. The diversity of dashboards and lack of standards for reporting and publishing data makes it challenging to aggregate and compare COVID-19 cases per county. Each dashboard has a different temporal and spatial unit of analysis that makes it difficult, and often-times impossible, to understand the trends of cases and any important cluster of infections. For instance, while 16 counties provide weekly information, 27 counties report daily cases. Citrus County only reports monthly cases (18). School-specific case surges may require unique mitigation strategies that may otherwise not apply to another institution, e.g., a measured targeted for a small rural elementary school may not be the same as a solution targeted for a large metropolitan high school. Importantly, all existing dashboards present only absolute case numbers without any contextual denominators such as the number of individuals at each school or enrolled at each county school district. None of current dashboards provides any contextual or demographic information such as age, sex, race, and vaccination status of students or staff.

**Table 1:** Public dashboards of COVID-19 cases in the florida public school system^1^.

**County**	**No. of schools**	**Dashboard**	**Data quality** ^2^						**Graphic quality**		
			**Level of aggregation**			**Full timeline (08/01/2021-05/23/2022)**	**Timeliness**	**Metadata** ^3^	**Graphic visualization**	**Filter**	**Cleanliness**
			**Temporal granularity**	**Spatial granularity**	**Distinctive information of student/staff**						
Alachua (32)	54	Y	Week	School	Y	Y	Weekly	N	Y	Y	Y
Baker	9	N	-	-	-	-	-	-	-	-	-
Bay (33)	41	Y	Week	County	Y	N	Weekly	N	Y	N	Y
Bradford	10	N	-	-	-	-	-	-	-	-	-
Brevard (34)	101	Y	Week	County	Y	N^4^	Weekly	N	N	N	Y
Broward (35)	301	Y	Day	School	Y	Y	Daily	Y	Y	Y	Y
Calhoun	7	N	-	-	-	-	-	-	-	-	-
Charlotte (19)	23	Y	Day	School	N	Y	Daily	Y	Y	Y	Y
Citrus (18)	22	Y	Month	School	Y	N	Stopped^5^	N	Y	N	Y
Clay (23)	48	Y	Week	County	Y	Y	Weekly	N	N	N	N
Collier (36)	61	Y	Day	School	Y	Y	Weekly	Y	N	Y	N
Columbia (37)	17	N^6^	Day	County	N	N	Stopped	N	N	N	N
DeSoto (20)	487	Y	Day	School	Y	N	Daily	N	N	N	N
Dixie	9	N	-	-	-	-	-	-	-	-	-
Duval (38)	9	Y	Week	School	Y	N	Weekly	Y	Y	N	N
Escambia	184	N	-	-	-	-	-	-	-	-	-
Flagler (39)	55	Y	Day	School	Y	Y	Stopped^7^	Y	N	N	N
Franklin	14	N	-	-	-	-	-	-	-	-	-
Gadsden (40)	6	Y	Day	School	Y	N	Stopped^8^	N	N	N	N
Gilchrist (41)	14	Y	Week	School	Y	Y	Stopped^9^	N	N	N	N
Glades (42)	8	Y	Week	School	Y	N	Stopped^10^	N	N	N	N
Gulf	9	N	-	-	-	-	-	-	-	-	-
Hamilton	6	N	-	-	-	-	-	-	-	-	-
Hardee (43)	6	Y	Day	School	Y	N	Daily	N	N	N	N
Hendry	11	N	-	-	-	-	-	-	-	-	-
Hernando	15	N	-	-	-	-	-	-	-	-	-
Highlands	29	N	-	-	-	-	-	-	-	-	-
Hillsborough (44)	20	Y	Day	School	Y	Y	Daily	N	Y	Y	Y
Holmes	274	N	-	-	-	-	-	-	-	-	-
Indian River (45)	9	Y	Day	School	Y	N	Stopped^11^	N	Y	N	N
Jackson	26	N	-	-	-	-	-	-	-	-	-
Jefferson	14	N	-	-	-	-	-	-	-	-	-
Lafayette	6	N	-	-	-	-	-	-	-	-	-
Lake (46)	6	Y	Day	School	Y	N	Daily	Y	Y	Y	Y
Lee (47)	55	Y	Day	School	Y	Y^12^	Daily	Y	Y	N	N
Leon (48)	101	N^13^	Day	School	Y	N	Stopped	N	Y	N	Y
Levy	48	N	-	-	-	-	-	-	-	-	-
Liberty (49)	15	N^14^	Week	County	-	N	Stopped	N	N	N	N
Madison	7	N	-	-	-	-	-	-	-	-	-
Manatee (50)	11	Y	Day	School	Y	N	Daily	Y	N	N	N
Marion (24)	65	Y	Week	County	Y	Y	Weekly	N	N	N	N
Martin (51)	52	Y	Day	School	Y	Y	Stopped^15^	Y	Y	Y	Y
Miami-Dade (21)	26	Y	Day	School	Y	N	Daily	N	Y	N	Y
Monroe (52)	20	Y	Week	School	Y	Y	Weekly	N	Y	Y	Y
Nassau (53)	19	Y	Week	School	Y	Y	Stopped^16^	N	N	N	N
Okaloosa (54)	42	Y	Week	School	N	Y	Stopped^17^	Y	Y	N	N
Okeechobee (55)	11	Y	Week	School	Y	N	Stopped^18^	Y	Y	Y	Y
Orange (56)	236	Y	Day	School	Y	Y	Daily	Y	Y	Y	Y
Osceola (57)	75	Y	Week	School	Y	Y	Weekly	N	N	N	N
Palm Beach (58)	204	Y	Day	School	Y	Y	Daily	N	Y	Y	Y
Pasco (59)	95	Y	Day	School	Y	N	Daily	Y	N	N	N
Pinellas (60)	133	Y	Day	School	Y	Y	Daily	Y	Y	Y	N
Polk (61)	139	Y	Day	School	Y	N	Daily	N	Y	Y	Y
Putnam (62)	19	Y	Day	School	Y	N	Daily	Y	N	N	N
Santa Rosa (63)	44	Y	Day	County^19^	Y	N	Daily	N	Y	N	N
Sarasota	45	N	-	-	-	-	-	-	-	-	-
Seminole (64)	36	Y	Week	School	N	N	Weekly	N	Y	N	Y
St. Johns (65)	53	Y	Day	School	Y	Y	Daily	N	N	N	N
St. Lucie (66)	67	Y	Day	School	Y	Y	Daily	N	Y	Y	Y
Sumter (67)	11	Y	Week	School	Y	Y	Stopped^20^	N	N	N	N
Suwanee	11	N	-	-	-	-	-	-	-	-	-
Taylor	8	N	-	-	-	-	-	-	-	-	-
Union	7	N	-	-	-	-	-	-	-	-	-
Volusia (68)	76	Y	Day	School	Y	Y	Daily	N	Y	Y	Y
Wakulla (69)	13	Y	Day	School	Y	N	Stopped^21^	N	N	N	N
Walton	18	N	-	-	-	-	-	-	-	-	-
Washington	8	N	-	-	-	-	-	-	-	-	-

^1^*Data current as of May 26, 2022*.

^2^*Due to the lack of consistent longitudinal data, we cannot measure some attributes of data quality such as Fidelity, Reliability, Sustainability, and Transparency*.

^3^*The Metadata category comprises of the following attributes: Introduction to the Data, Source of Data, Publishing Schedule*.

^4^*Brevard County provided full timeline information until February 20, 2022. However, they decided to post only the last seven days of positive cases as of February 21, 2022*.

^5^*Citrus County stopped providing information from November 1, 2022*.

^6^*Columbia County does not provide any dashboard when we revisited on May 26, 2022, but data and graphic quality indicators follow observations from February 1, 2022*.

^7^*Flagler County stopped providing information from March 2, 2022*.

^8^*Gadsden County stopped providing information from February 21, 2022*.

^9^*Gilchrist County stopped providing information from February 21, 2022*.

^10^*Glades County stopped providing information when we revisited on May 26, 2022*.

^11^*Indian River County stopped providing information from April 29, 2022*.

^12^*Lee County provides only aggregated daily number for the full timeline*.

^13^*Leon County does not provide any dashboard as of May 11, 2022, but data and graphic quality indicators follow observations from February 1, 2022*.

^14^*Liberty County does not provide any dashboard as of May 26, 2022, but data and graphic quality indicators follow observations from February 1, 2022*.

^15^*Martin County stopped providing information from April 29, 2022*.

^16^*Nassau County stopped providing information from February 4, 2022*.

^17^*Okaloosa County stopped providing information from March 28, 2022*.

^18^*Okeechobee County stopped providing information from January 14, 2022*.

^19^*Santa Rosa County provides school-specific information on positive student cases (although not updated), but does not do so for positive staff cases*.

^20^*Sumter County stopped providing information from November 3, 2022*.

^21^*Wakulla County stopped providing information as of March 28, 2022*.

Thirty-eight county school boards (57% of counties) do not provide any dashboard to trace students and staff. Pathogens are rarely contained within man-made administrative geographic boundaries, especially when dealing with a pandemic. Informed decisions require county school boards have accurate information about their neighboring counties. For example, although Charlotte County (19) provides an informative dashboard, the contiguous Sarasota County does not publicly post data that would allow administrators to account for the neighboring county's spillover infections.

Several dashboards do not provide information on COVID-19 cases since the beginning of the 2021-2022 school year in a continuous manner, and each county has a different “update-and-delete” schedule. For example, Citrus County decided to stop reporting after late October 2021 (18). The latter policy prevents residents of this county to track how many students and staff were infected by the omicron variant spreading throughout Florida since December 2021. Importantly, 7 dashboards only indicate the cases of the current day [e.g., DeSoto County (20)], while others only show the cases of the current month [e.g., Miami-Dade (21)]. To evaluate the impact of specific county health policies or perform any type of program evaluation, it is important to trace the number of infections across the span of the academic year with timely data refreshers. To illustrate, in September 2021, the newly appointed Florida Surgeon General enacted a policy that did not require exposed asymptomatic students to quarantine (22). With the limited (and deleted) information from the available dashboards, it is impossible to conduct any meaningful real-time policy evaluation.

Our environmental scan revealed it is often difficult to extract COVID-19 case information from current dashboards due to their poor design, inadequate graphical user interface, or lack of functional export tools. Nine counties only provide case data in downloadable PDF or Google Doc^®^ files. Data provided across separate files impedes systematic or automated data collection (e.g., use of application programming interface software) and hampers any efficient evaluation. Moreover, there is heterogeneity on how the case data is reported on the visual interface of the dashboards. Currently, 20 counties only present the public with raw case numbers rather than presenting any accompanying visual aid. A combination of interactive graphics and export capabilities would help communicate information better to the public and allow interested parties to extract relevant data. In addition, it is important to provide a simple display of essential data that does not require extensive navigation. Some counties [Clay (23) and Marion (24)] provide a webpage with multiple links corresponding to COVID-19 cases during specific time periods. Burdensome interfaces like these are undesirable because they can hinder users from accessing important COVID-19 data when they seek more direct displays. Many of the published dashboards do not allow users to filter the case data (e.g., type of school), which limits users on the information available for analysis and might lead to inaccurate inference from the aggregated (non-filtered) data.

## Discussion

### Recommendations for improvement

Given our review of the existing county school board's COVID-19 dashboards in Florida, we aim to pose several policy recommendations and to discuss future directions in meaningful measures for the evolving pandemic. First, state-level and federal-level support is essential to improve current surveillance. We observed significant heterogeneity among counties, both in availability and framework of current platforms. While some counties established excellent dashboards with common features to inform public health, most counties did not provide any data or low-quality data. We observed excellent dashboards in those counties with highest median income in the State (25). This finding might highlight how counties might not have enough infrastructure to establish and maintain a data reporting system on their own. Institutions like state Departments of Health and Departments of Education could collaborate in supporting the development of an effective dashboard template and distribute it to each county. Even though this approach could help overcome resource-based restrictions, we note that possible resistance of local government officials or school boards is an important factor that requires additional oversight.

How to manage the COVID-19 pandemic has become heavily politicized in the United States. Political ideology has become one of the critical factors that affect individual's health policy compliance during the pandemic (26–28). Moreover, American federalism has hindered unified and coherent national policy by the federal government, as partisan polarization has driven each governor to conduct divergent policy (29, 30). Florida state politics have exemplified some of these attitudes by the decision-making of leaders and administrators on enforcement of restrictive mitigation measures of the pandemic, such as the aforementioned emergency rule by the Surgeon General (22) and strong stances against mask mandates. For instances when state leaders are resistant to implement standardized data collection and reporting systems across school boards, it is important to create multi-county coalitions. To preserve resources, geographically contiguous counties could join forces to create a single platform to report or feed information bidirectionally.

Significantly, there is scarcity of valuable contextual data for cases reported among the functional dashboards. Data elements should include information such as race, gender, and vaccination status, as well as provide denominators relevant to the unit of analysis reported. Even among the dashboards that provide daily cases of students and staff per school from the beginning of the fall 2021 semester, it is difficult to understand potential clusters of infection without additional characteristics of students and staff. Augmenting the dashboards to provide more detailed information, preferably with a filtering mechanism, would allow researchers and policy makers to better evaluate the effect of policies and provide more timely feedback. As current dashboards with limited information do not raise privacy concerns, including more detailed information can introduce privacy issues. Future dashboards should ensure compliance with law requirements and pursue a balance between information and privacy. Furthermore, it is critical to provide a user-friendly and accessible dashboard to parents and the community at large. The dashboards with a well-visualized graph enable residents to check them regularly and react to any COVID-19 surges happening in their school community. Dashboards with tedious processes for data extraction can be a hinderance to the public in their need to stay informed.

Whether the information provided in simple infection case dashboards can continue to provide helpful information has come into question lately. Even after extensive improvements to public dashboards, the rapidly changing landscape of the pandemic necessitates frequent updating of these systems. Moving beyond simple administrative reporting of school cases, some epidemiologists have called for the need for surveillance using random sampling in light of increased home-based testing and other factors that prevent a meaningful understanding of tracked cases (31). While random sampling would allow for better inferences that are currently not possible with the existing data sources, this approach would incur in significant costs. Additional work is needed to understand end-users' attitudes and perspectives on the use of available dashboards as well as levels of utilization of these systems in the real world.

## Conclusion

Overall, the public dashboards that track cases of students and staff in the Florida public school system are inadequate. In addition to a considerable number of counties not reporting data, those with functional dashboards are often limited in their functionalities that preclude any meaningful evaluation of public health initiatives. Administrative oversight is needed to improve the current systems if they are to provide helpful and timely data to residents.

## Data availability statement

The original contributions presented in the study are included in the article/supplementary material, further inquiries can be directed to the corresponding author/s.

## Author contributions

JH and HJ: study conception and design. JH, HJ, and JQ: acquisition, analysis, or interpretation of data, drafting of the manuscript, and critical revision of the manuscript for important intellectual content. HJ and JQ: statistical analysis. All authors reviewed the results and approved the final version of the manuscript.

## Conflict of interest

JH reports receiving funding from Merck, Sharp & Dohme for a research project unrelated to the content of this manuscript. The remaining authors declare that the research was conducted in the absence of any commercial or financial relationships that could be construed as a potential conflict of interest.

## Publisher's note

All claims expressed in this article are solely those of the authors and do not necessarily represent those of their affiliated organizations, or those of the publisher, the editors and the reviewers. Any product that may be evaluated in this article, or claim that may be made by its manufacturer, is not guaranteed or endorsed by the publisher.
